# Methylation status of TK1 correlated with immune infiltrates in prostate cancer

**DOI:** 10.3389/fgene.2022.899384

**Published:** 2022-08-11

**Authors:** Chenming Zhang, Sicheng Ma, Xiaohui Hao, Zulong Wang, Zixue Sun

**Affiliations:** ^1^ The Second Clinical Medical School, Henan University of Chinese Medicine, Zhengzhou, China; ^2^ Andrology Department, The First Affiliated Hospital of Henan University of Chinese Medicine, Zhengzhou, China; ^3^ Reproductive Medicine Department, Henan Province Hospital of Traditional Chinese Medicine, Zhengzhou, China

**Keywords:** prostate cancer, thymidine kinase 1, prognostic biomarker, immune infiltrates, DNA methylation

## Abstract

TK1 is overexpressed in numerous cancers and is associated with to a poor prognosis. However, the relationship between methylation status of TK1 and Immune Infiltrates in Prostate Cancer (PCa) is unknown. The goal of this study was to use comprehensive bioinformatic analyses to elucidate the involvement relationship between methylation status of TK1 and Immune Infiltrates in PCa. TK1 mRNA expression and methylation data in PCa were investigated via GEPIA, TIMER, and UALCAN coupled with MEXPRESS data resources. We employed the LinkedOmics data resource to determine the signaling cascades linked to TK1 expression. Single-cell analysis was performed using the CancerSEA data resource. GeneMANIA and CancerSEA were used to analyze the correlation between TK1 and TK1 coexpressed genes. In addition, TIMER and TISIDB were adopted to assess tumor-invading immune cells and immunomodulators. CTD was utilized to detect the drugs acting on TK1. This study found that TK1 was overexpressed in PCa, and its contents were linked to tumor stage and prognosis. Genes co-expressed with TK1 were enriched in cascades involved in the ribosome, cell cycle, oxidative phosphorylation, DNA replication, oocyte meiosis, and the proteasome. The expression of TK1 along with its methylation status was found to be linked to tumor-invading immune cells, as well as PCa immunomodulators. We also examined the prospect of employing TK1 as a possible target for PCa therapy. This work provides the clinical value of TK1 hypermethylation in PCa and highlights new insights into its novel immunomodulatory functions.

## Introduction

Prostate cancer (PCa) is one of the most frequent cancers in men, with approximately 1.1 million reported cases worldwide. (Luszczak et al., 2020). Furthermore, PCa has surpassed bladder and kidney cancers in terms of incidence and mortality (Mayer et al., 2001). PCa progresses in a multistep process and is thought to progress from precancerous or intraepithelial neoplasia of the prostate to locally aggressive, metastatic, and hormone-resistant disease (Khan et al., 2008). Several factors can increase the risk of PCa, such as family factors, baldness, gonorrhea, and smoking (Rizzardi et al., 2014; Rao et al., 2015; Ju-Kun et al., 2016), and some unchangeable risk factors include age, race, and family history (Facompre & El-Bayoumy, 2009). Crosstalk between tumor cells the tumor microenvironment (TME) provides new perspectives for understanding the molecular drivers of PCa onset, metastasis, and recurrence (Matos et al., 2021; Melo et al., 2021). However, the molecular pathology of PCa is complex (Xiao et al., 2018). In addition to somatic mutations and chromosomal abnormalities that result in the dysregulation of tumor suppressor genes and oncogenes, changes in the expression of growth factors and their receptors are also involved in the pathogenesis of PCa (Cazares et al., 2010).

Thymidine kinase 1 (TK1) is an enzyme in the DNA salvage pathway, that involved in DNA synthesis and damage through regenerating thymidine. Through facilitated diffusion, thymidine is transferred from the extracellular space to the cell membrane. Where it is converted into its monophosphate form (dTMP) by cytoplasmic TK1 (Bello, 1974; Johnson et al., 1982). As early as the 1960s, the activity of TK1 in tumors was found to be elevated (Weber & Lea, 1966). Numerous studies have further demonstrated that elevated TK1 levels have occur in the serum of many different types of cancers, including some of the most common cancers of the lung cancer, colon cancer, and breast cancer (Alegre et al., 2012; Jagarlamudi & Shaw, 2018; Topolcan & Holubec, 2008; Zhou et al., 2013). Recent findings suggest that intracellular TK1 is correlated with cancer cell invasion and progression, along with its signature role in cancer cell proliferation (Alshabi et al., 2019). The molecular mechanisms of intracellular TK1 overexpression have not been established, and its association with cancer progression has not been sufficiently explored. TK1 overexpression may not only be a by-product of cancer cell processes but rather, part of a selective process that aids cancer cell progression. Tumor growth supported by TK1 has been demonstrated in lung and breast cancer cell lines (Bitter et al., 2020). However, the function of TK1 in PCa remains unclear.

The control and adaptability of virtually all biological processes involve postsynthesis chemical modification of three classes of fundamental macromolecules: DNA, RNA and proteins. One of the most abundant modifications, methylation, is widespread throughout all kingdoms of life, and involves an alkylation reaction whereby a methyl group replaces a hydrogen atom. Methylation is catalysed by methyltransferases (‘writers’), which use S-adenosylmethionine (SAM) as the methyl donor; writers cooperate with dedicated ‘erasers’ (demethylases) and methyl ‘readers’. The importance of the various methylation pathways is highlighted by the fact that their deregulation is linked to many diseases.

Here, we used several available data resources to conduct a comprehensively analyze TK1 in PCa. We found that TK1 was remarkably overexpressed in PCa and that TK1 content was linked to tumor prognosis in individuals with PCa. TK1 coexpressed genes were grouped into cascades involving the cell cycle, oxidative phosphorylation, DNA replication, oocyte meiosis, and the proteasome. In PCa, the content of TK1 and its methylation status were linked to tumor-invading immune cells and immunomodulators. In addition, we explored the possibility of using TK1 as a treatment target for PCa. This findings of this study highlight a novel immunomodulatory function of TK1 hypermethylation in PCa.

## Materials and methods

### Analysis of gene expression differences

We used the UALCAN data resource (http://ualcan.path.uab.edu/) (Chandrashekar et al., 2017) to study the expression of TK1 mRNA in tumor tissues and in normal tissues and between different nodal metastasis statuses of PCa tissues, the GEPIA data resource (http://gepia2.cancer-pku.cn/) (Tang et al., 2019) was utilized to investigate the differential expression of TK1 in several cancer species with significantly different TKl expression, The HPA data resource (Human Protein Atlas, www.proteinatlas.org) (Thul & Lindskog, 2018) was used to determine the differential protein expression levels of TK1 between PCa and nontumorous tissues.

### Prognostic value of TK1 and pathologic parameter correlation

We used the GEPIA data resource to determine the relationship between TK1 expression with overall survival (OS) and disease-free survival (DFS). Single- and multifactor Cox regression analyses for OS and DFS were performed using the survivor package in R (v3.2-10).

### Methylation analysis

We analyzed the methylation levels of the TK1 promoter in normal and cancerous tissues at different stages using the UALCAN data resource. The methylation levels of TK1 CpG islands in TK1 in PCa were then analyzed using the MEXPRESS data resource (https://mexpress.be) (Koch et al., 2019).

### Coexpressed gene analysis and functional enrichment analyses

We analyzed the list of genes coexpressed with TK1 in PCa using the LinkedOmics data resource (http://www.linkedomics.org/login.php) (Vasaikar et al., 2018). The gene and protein interaction networks of TK1 were constructed using GeneMANIA data resource (http://www.genemania.org) (Warde-Farley et al., 2010) and STRING data resource (https://string-db.org/) (Szklarczyk et al., 2021), respectively. The Gene Ontology (GO) and Kyoto Encyclopedia of Genes and Genomes (KEGG) pathway enrichment analyses were also used for the LinkedOmics data. To elucidate the function of TK1 in PCa tissues, we carried out a single-cell analysis using the CancerSEA data resource (http://biocc.hrbmu.edu.cn/CancerSEA/) (Yuan et al., 2019).

### Association of TK1 and E2F transcription factors in PCa

Human expression levels of E2Fs in PCa from The Cancer Genome Atlas (TCGA) data resource were estimated using the ggplot2 R package (v3.3.3).

### Tumor immune estimation analysis

Immune cell richness was estimated via the GSA R package (v1.34.0) algorithm (Hanzelmann et al., 2013). The correlation module was utilized to establish the relationship between TK1 RNA-seq expression profile data in PCa and immune cells, which included T cells, NK cells, Tfh (T follicular helper) cells, aDC (activated DC), macrophages, Tcm (T central memory) cells, B cells, cytotoxic cells, Th17 cells, DC, iDC (immature DC), mast cells, NK CD56bright cells, eosinophils, pDC (plasmacytoid DC), T helper cells, NK CD56dim cells, Th1 cells, Tem (T effector memory) cells, CD8 T cells, Tgd (T gamma delta) cells, neutrophils, Th2 cells, and Treg cells. The gene markers for immune cells also use gene modules associated with TK1 expression as referenced in previous studies (Bindea et al., 2013).

### Association of TK1 methylation with immune infiltrates in PCa

Our study analyzed the relationship between TK1 methylation and immunocytes and immunomodulators by the TISIDB data resource (http://cis.hku.hk/TISIDB) (Ru et al., 2019).

### Drug response linked to TK1 expression in PCa

To assess whether TK1 could be a treatment target for individuals with cancer, we obtained a targeted drug for TK1 from the CTD data resource (http://ctdbase.org/) (Davis et al., 2021). We then visualized them via the ggalluvial R package (v3.3.3) algorithm.

## Results

### Expression of TK1 in PCa

**FIGURE 1:**
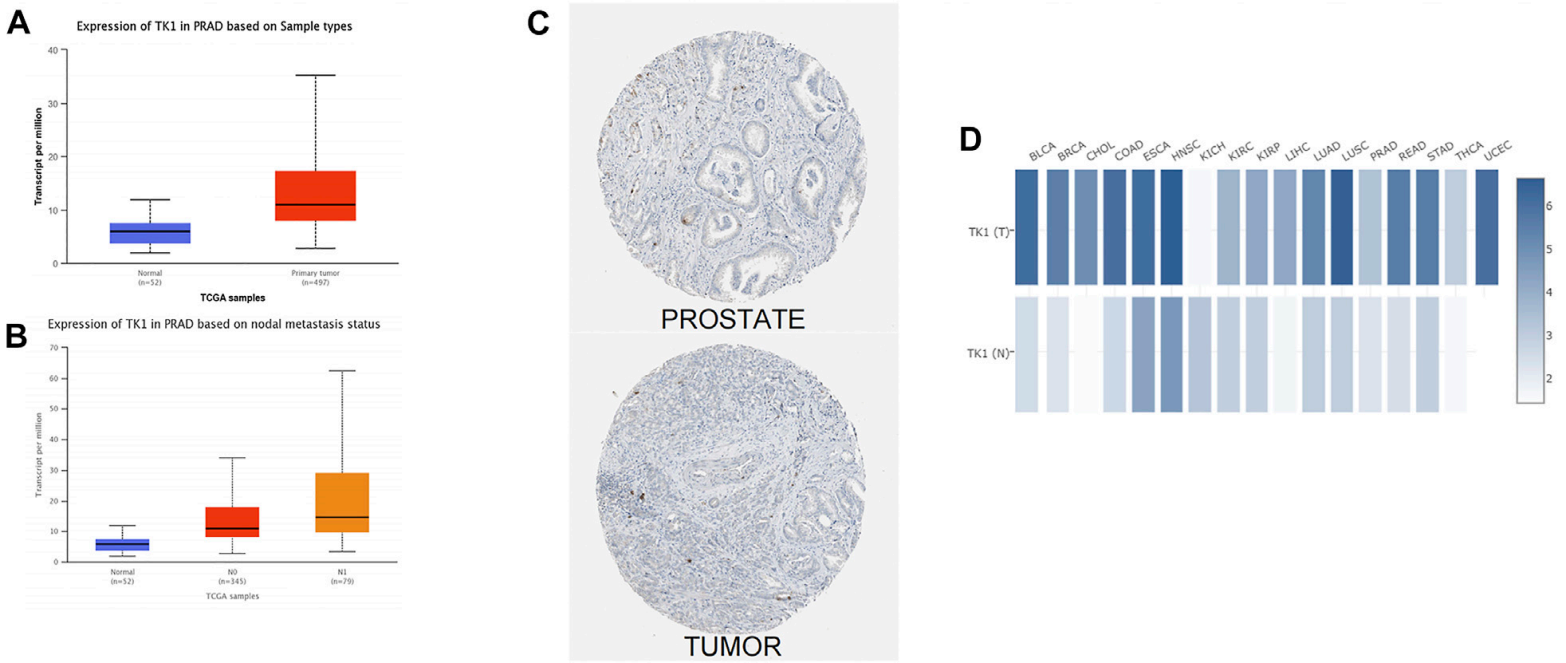
Overexpression of TK1 in PCa. **(A)** TK1 expression levels in various human malignancies were analyzed by TIMER. **(B)** UALCAN analyzed TK1 mRNA levels in tumor tissues and nontumorous tissues. **(C)** The expression of TK1 mRNA between different nodal metastasis statuses of PCa tissues was analyzed by UALCAN. **(D)** Differential protein expression of TK1 in tumor and nontumorous tissues in the HPA. (E) GEPIA analyzed high or low expression levels of TK1 in various malignant tumors.

The analysis of the UALCAN data resource further confirmed that expression of TK1 transcript in primary PCa was higher than that in paraneoplastic tissues (Figure 1A). In addition, TK1 presented strong positivity in PCa tissues with different nodal metastasis statuses, and this was in contrast TK1 levels to non-tumorous tissues (Figure 1B). Interactive analysis of the gene expression profiles also verified that TK1 levels were higher in PCa tumor tissues than in paraneoplastic tissues (Figure 1C). The GEPIA data resource confirmed these results (Figure 1D).

### The prognostic relevance of TK1 and TK1’s relationship with clinicopathological parameters in PCa

**FIGURE 2:**
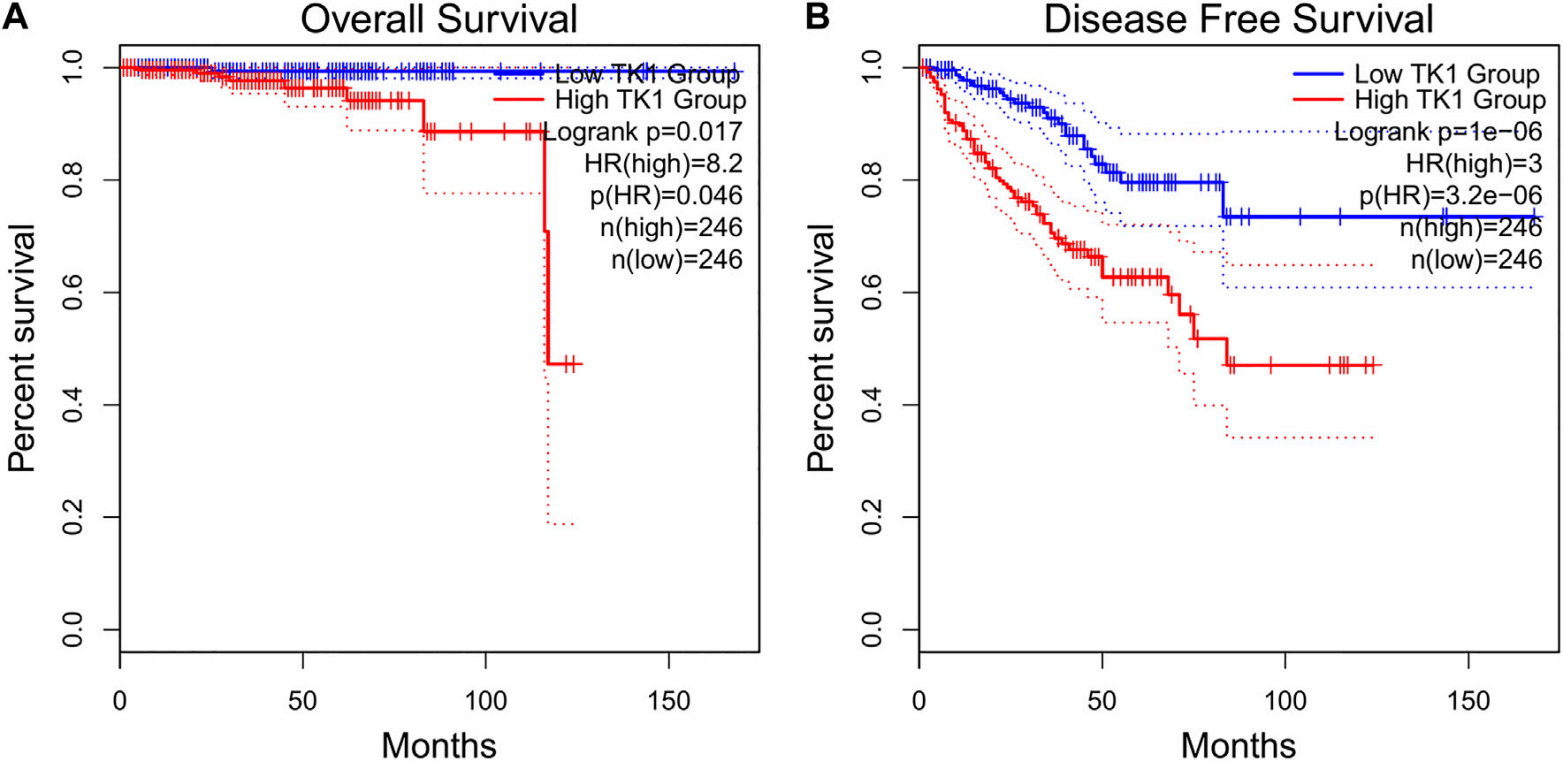
Prognostic value as a result of TK1 in PCa patients. **(A,B)** GEPIA profiling showed that PCa patients with high TK1 expression were associated with poorer OS. **(A)** and PFS **(B)** relative to those with low TK1 expression.

The baseline features of the patients are given in Table 1. Among the patients, 499 had both the clinical and genetic characteristics of PCa. The GEPIA assessment illustrated that high TK1 expression was significantly linked to poor OS (HR = 8.2, *p* = 0.046) and DFS (HR = 3, *p* = 3.2e-06) in individuals with PCa (Figures 2A,B). The univariate Cox data showed that OS was linked to the M stage, Gleason score, primary therapy outcome, and TK1 expression. (Table 2). Furthermore, the univariate Cox regression analysis showed that T stage, Gleason score, N stage, primary therapy outcome, and TK1 expression were associated with PFS. Lastly, the multivariate Cox regression revealed that the Gleason score, TK1 content, and primary treatment outcome were prognostic parameters in individuals with PCa (Table 3). These data indicate that there is an association between TK1 and a worsening prognosis.

**TABLE 1:** Relationship between TK1 expression and clinicopathological features in patients with PCa.

Characteristics	Low expression of TK1	High expression of TK1	*p* Value
N	249	250	
Age, n (%)			0.022
≤60	125 (25.1%)	99 (19.8%)	
>60	124 (24.8%)	151 (30.3%)	
Race, n (%)			0.781
Asian	5 (1%)	7 (1.4%)	
Black or African American	30 (6.2%)	27 (5.6%)	
White	207 (42.8%)	208 (43%)	
T stage, n (%)			<0.001
T2	118 (24%)	71 (14.4%)	
T3	124 (25.2%)	168 (34.1%)	
T4	3 (0.6%)	8 (1.6%)	
N stage, n (%)			0.007
N0	180 (42.3%)	167 (39.2%)	
N1	27 (6.3%)	52 (12.2%)	
M stage, n (%)			1.000
M0	225 (49.1%)	230 (50.2%)	
M1	1 (0.2%)	2 (0.4%)	
PSA (ng/ml), n (%)			0.158
<4	220 (49.8%)	195 (44.1%)	
≥4	10 (2.3%)	17 (3.8%)	
Gleason score, n (%)			<0.001
6	33 (6.6%)	13 (2.6%)	
7	146 (29.3%)	101 (20.2%)	
8	30 (6%)	34 (6.8%)	
9	39 (7.8%)	99 (19.8%)	
10	1 (0.2%)	3 (0.6%)	
Residual tumor, n (%)			0.003
R0	173 (37%)	142 (30.3%)	
R1	58 (12.4%)	90 (19.2%)	
R2	3 (0.6%)	2 (0.4%)	
Age, mean ± SD	60.53 ± 7.11	61.52 ± 6.49	0.104

**TABLE 2:** Univariate and multivariable Cox regression of TK1 expression for overall survival in PCa patients.

Characteristics	Total (n)	Univariate analysis		Multivariate analysis	
		Hazard ratio (95% CI)	P value	Hazard ratio (95% CI)	P value
≤60	224	Reference			
>60	275	1.577 (0.440-5.648)	0.484		
T stage	492				
T2	189	Reference			
T3&T4	303	3.294 (0.612-17.727)	0.165		
N stage	426				
N0	347	Reference			
N1	79	3.516 (0.778-15.896)	0.102		
M stage	458				
M0	455	Reference			
M1	3	59.383 (6.520-540.817)	<0.001	22.369 (0.681-734.908)	0.041
Gleason score	499				
6&7&8	357	Reference			
9&10	142	4.842 (1.206-19.436)	0.026	0.542 (0.084-3.502)	0.520
Primary therapy outcome	438				
PD&SD&PR	97	Reference			
CR	341	0.111 (0.022-0.552)	0.007	0.165 (0.023-1.175)	0.072
Residual tumor	468				
R0	315	Reference			
R1&R2	153	2.598 (0.696-9.694)	0.155		
PSA (ng/ml)	442				
<4	415	Reference			
≥4	27	10.479 (2.471-44.437)	0.001	4.829 (0.886-26.310)	0.069
TK1	499	2.226 (1.130-4.385)	0.021	1.980 (0.850-4.613)	0.113

**TABLE 3:** Univariate and multivariable Cox regression of TK1 expression for progression-free survival in PCa patients.

Characteristics	Total (N)	Univariate analysis		Multivariate analysis	
		Hazard ratio (95% CI)	*p* Value	Hazard ratio (95% CI)	*p* Value
Age	499				
≤60	224	Reference			
>60	275	1.302 (0.863-1.963)	0.208		
T stage	492				
T2	189	Reference			
T3&T4	303	3.785 (2.140-6.693)	<0.001	1.442 (0.690-3.017)	0.331
N stage	426				
N0	347	Reference			
N1	79	1.946 (1.202-3.150)	0.007	0.812 (0.463-1.423)	0.466
M stage	458				
M0	455	Reference			
M1	3	3.566 (0.494-25.753)	0.208		
Gleason score	499				
6&7&8	357	Reference			
9&10	142	4.590 (3.038-6.934)	<0.001	2.040 (1.160-3.588)	0.013
Primary therapy outcome	438				
PD&SD&PR	97	Reference			
CR	341	0.151 (0.099-0.231)	<0.001	0.283 (0.161-0.499)	<0.001
Residual tumor	468				
R0	315	Reference			
R1&R2	153	2.365 (1.566-3.570)	<0.001	1.041 (0.609-1.781)	0.883
PSA (ng/ml)	442				
<4	415	Reference			
≥4	27	4.196 (2.095-8.405)	<0.001	1.990 (0.894-4.432)	0.092
TK1	499	1.862 (1.506-2.302)	<0.001	1.486 (1.119-1.975)	0.006

### DNA methylation of TK1 and PCa prognosis

**FIGURE 3:**
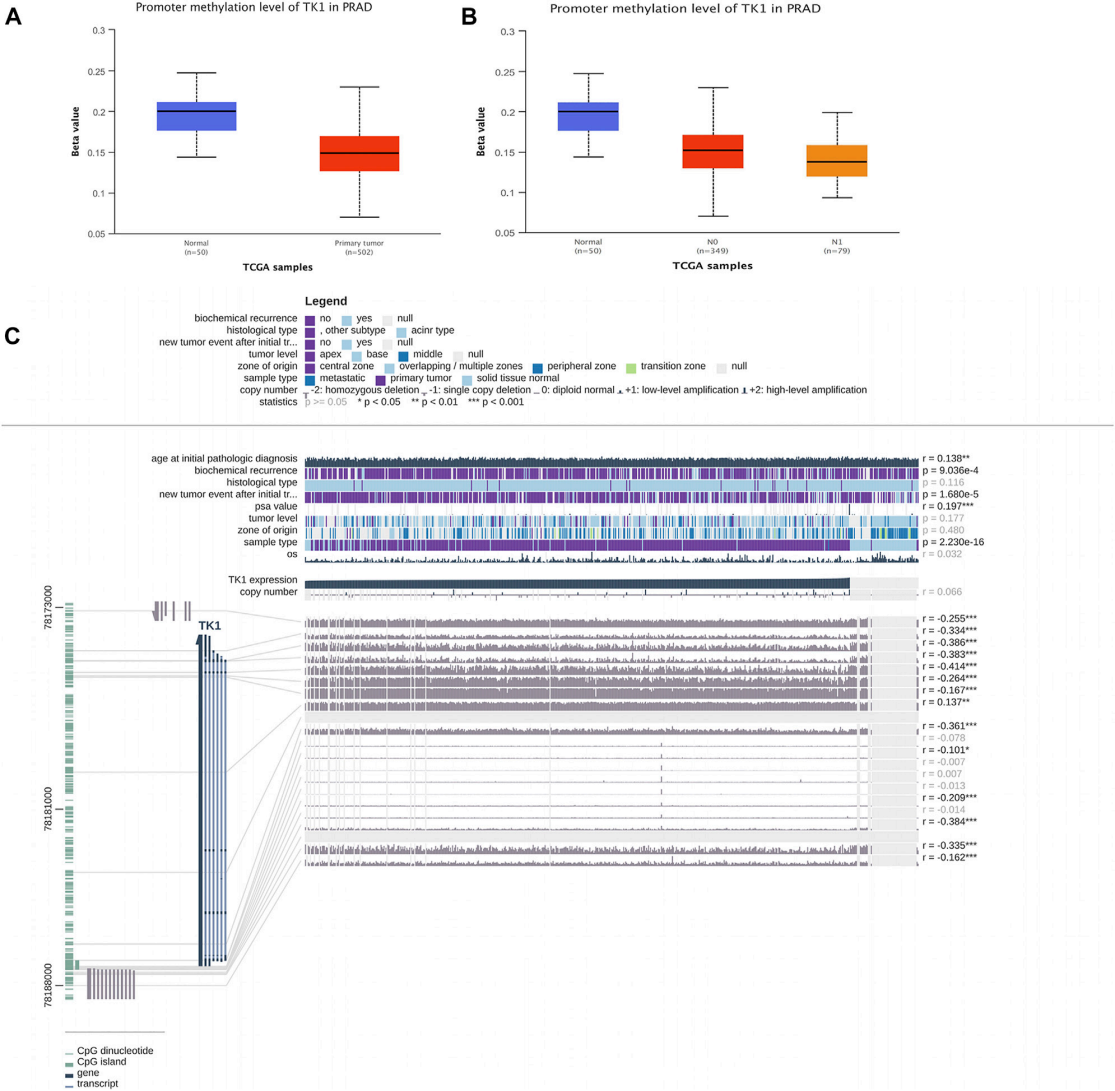
DNA methylation level of TK1 in PCa. **(A)** UALCAN data resource showing promoter methylation levels of TK1 in normal and PCa tissues. **(B)** UALCAN data resource offering promoter methylation levels of TK1 in PCa tissues with different nodal metastasis statuses. **(C)** Relationship between TK1 expression and PCa and methylation around CpG islands and promoter regions.

TK1 expression was negatively correlated with the methylation of CpG islands and promoter regions. In PCa cancer tissues, the DNA methylation level of TK1 was remarkably lower than that in non-tumorous samples. (Figure 3A). Furthermore the DNA methylation level of N1 was lower than that in N0 in PCa tissues with different nodal metastasis statuses, while the DNA methylation level of N0 was lower than that in nontumorous tissues (Figure 3B). The expression of TK1 was negatively correlated with methylation around CpG islands and promoter regions (Figure 3C). Thirteen aberrant methylation sites (cg10456035, r = -0.255; cg20740903, r = -0.334; cg25069807, r = -0.386; cg21519872, r = -0.414; cg08115732, r = -0.264; cg00715343, r = -0.167; cg18767057, r = -0.137; cg03291825, r = -0.361; cg26206461, r = -0.101; cg07314523, r = -0.209; cg22061523, r = -0.384; cg02441982, r = -0.335; cg21940220, r = -0.162), and the observed Pearson correlation coefficients are indicated on the right. Here, DNA methylation of the TK1 promoter may further support the TK1 overexpression in PCa.

### Gene and functional enrichment analysis of TK1 coexpression

**FIGURE 4:**
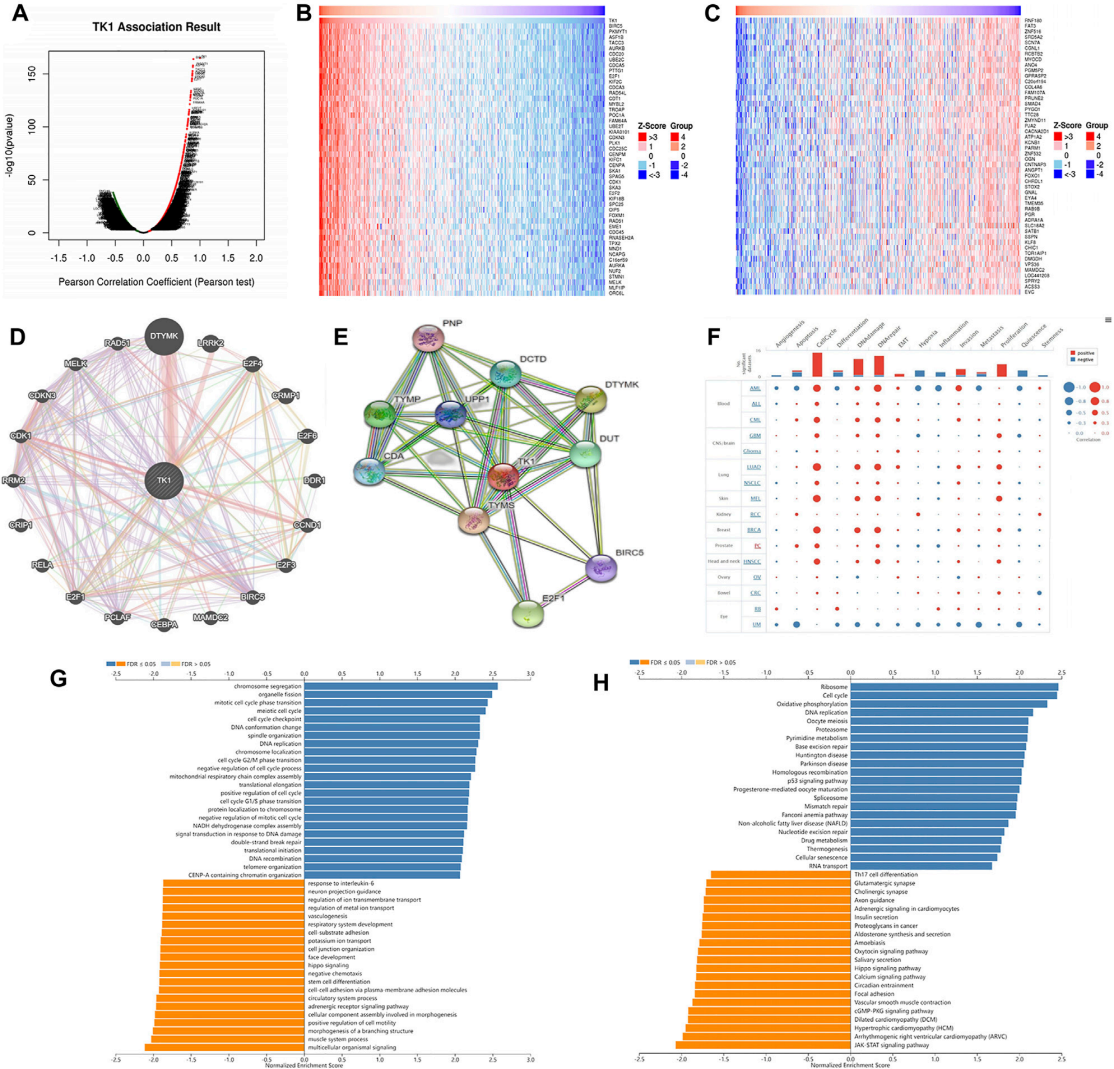
Gene and functional enrichment analysis of TK1 coexpression. **(A)** Volcano map of TK1 coexpression in PCa according to the LinkedOmics data resource. **(B,C)** Heatmap of the top 50 genes positively **(B)** and negatively **(C)** linked to TK1 are shown. **(D)** Gene network associated with TK1, analysis by GeneMANIA. **(E)** Interaction network map between proteins encoded by TK1 genes plotted with STRING. **(F)** Single-cell analysis of the CancerSEA data resource indicates that TK1 is mainly involved in the cell cycle, DNA repair, and apoptosis. **(G,H)** TK1 coexpressed genes were annotated on LinkedOmics via Gene Ontology (GO) assessment **(G)** and Kyoto Encyclopedia of Genes and Genomes (KEGG) pathway analysis **(H)**.

**FIGURE 5:**
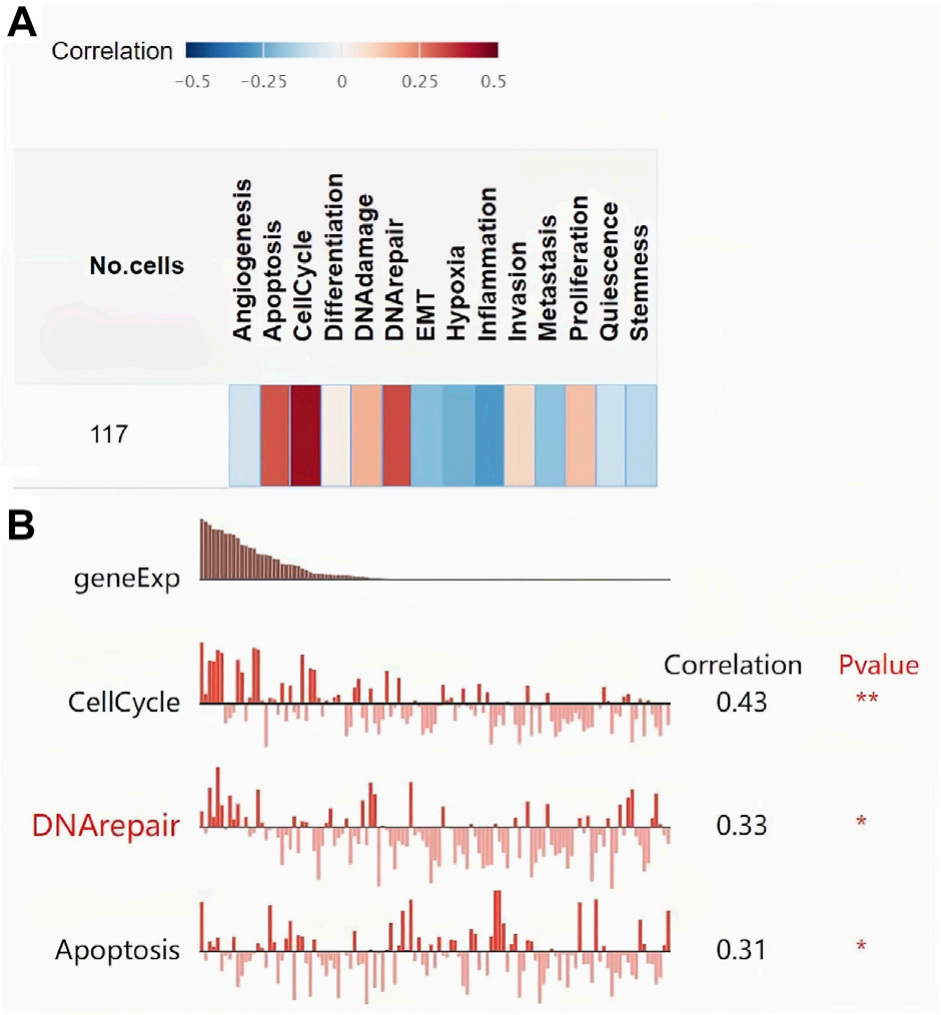
Single-cell analysis of the functional characteristics of TK1 in prostate cancer. **(A)** Correlation between TK1 and 14 cell functions. **(B)** The screened functional state with significant indigenous correlation with TK1.

Figure 4 Ashows the coexpression trend of TK1. The heat map contains the top 50 positively correlated genes along with the negative genes linked to TK1 (Figures 4B,C). The genes along with the protein-protein interaction networks created via GeneMANIA and STRING revealed that 20 potential target genes (DTYMK, LRRK2, CRMP1, DDR1, CCND1, BIRC5, MAMDC2, CEBPA, PCLAF, E2F1/3/4/6, RELA, CRIP1 RRM2, CDK1, CDKN3, MELK, RAD51) and ten potential target proteins (PNP, TYMP, CDA, DCTD, UPP1, DTYMK, TYMS, DUT, BIRC5, E2F1) that interacted with TK1 (Figures 4D,E). To further characterize the function of TK1 in PCa, we conducted a single-cell analysis using CancerSEA. The results illustrated that TK1 was mainly involved in the cell cycle, DNA repair, and apoptosis (Figure 4F). The annotation of the GO terms showed that the co-expressed genes of TK1 mainly participated in chromosome segregation, organelle fission, DNA replication, mitotic cell cycle phase transition, meiotic cell cycle, DNA conformation change, spindle organization, chromosome localization, the cell cycle G2/M phase transition, harmful modulation of the cell cycle process, mitochondrial respiratory chain complex assembly, translational elongation, positive modulation of the cell cycle, the cell cycle G1/S phase transition, protein localization to chromosome, negative regulation of mitotic cell cycle, NADH dehydrogenase complex assembly, signal transduction in response to DNA damage, etc. (Figure 4G). The KEGG pathway analysis indicated enrichment in the ribosome, cell cycle, oxidative phosphorylation, DNA replication, oocyte meiosis, proteasome, pyrimidine metabolism, base excision repair, Huntington disease, Parkinson disease, homologous recombination, p53 signaling cascade, nonalcoholic fatty liver disease, progesterone-mediated oocyte maturation, spliceosome, mismatch repair, Fanconi anemia cascade, nucleotide excision repair, etc. (Figure 4H). Single-cell analysis of the functional characteristics of TK1 in prostate cancer showed that TK1 expression positively correlated with Cell cycle (*p* = 0.001), DNA repair (*p* = 0.003), Apoptosis (*p* = 0.005) Figure 5

### Association of TK1 and E2F transcription factors in PCa

**FIGURE 6:**
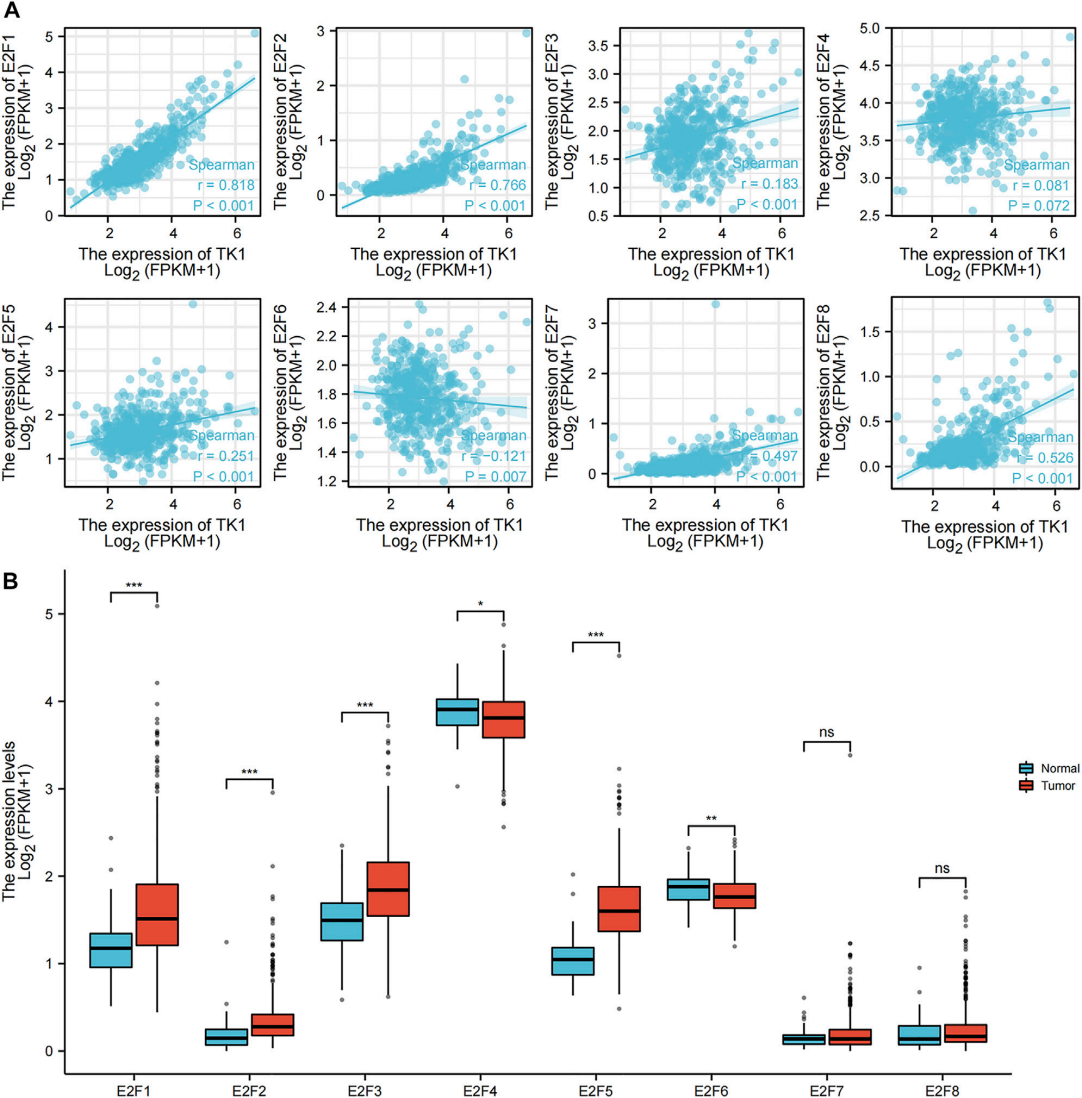
Association of TK1 and E2Fs in PCa. **(A)** Molecular correlation analysis of TK1 and E2Fs. **(B)** Human expression levels of E2Fs in PCa from The Cancer Genome Atlas (TCGA) data resource were estimated by the ggplot2 R package (v3.3.3) algorithm. (*<0.05, **<0.01, ***<0.001).

The association between TK1 and E2F transcription factors was investigated by using a ggplot2 R package (v3.3.3) algorithm analysis (Figure 6). E2F1 (r = 0.818, *p* = 0.001), E2F2 (r = 0.766, *p* = 0.001), E2F3 (r = 0.183, *p* = 0.05), E2F4 (r = 0.081, *p* = 0.072), E2F5 (r = 0.251, *p* = 0.001), E2F7 (r = 0.497, *p* = 0.001), and E2F8 (r = 0.526, *p* = 0.001) were all positively linked to TK1 expression. E2F6 (r = -0.121, *p* = 0.007) was negatively correlated with TK1 expression (Figure 6A). The results of the ggplot2 package (v3.3.3) algorithm analysis further confirmed that E2F1, E2F2, E2F3 and E2F5 expression were higher in primary PCa cancer tissues than in precancer tissues; however, E2F4 and E2F6 were lower in primary PCa tissues than in precancerous tissues (Figure 6B).

### Relationship of TK1 with immune cell invasion in PCa

**FIGURE 7:**
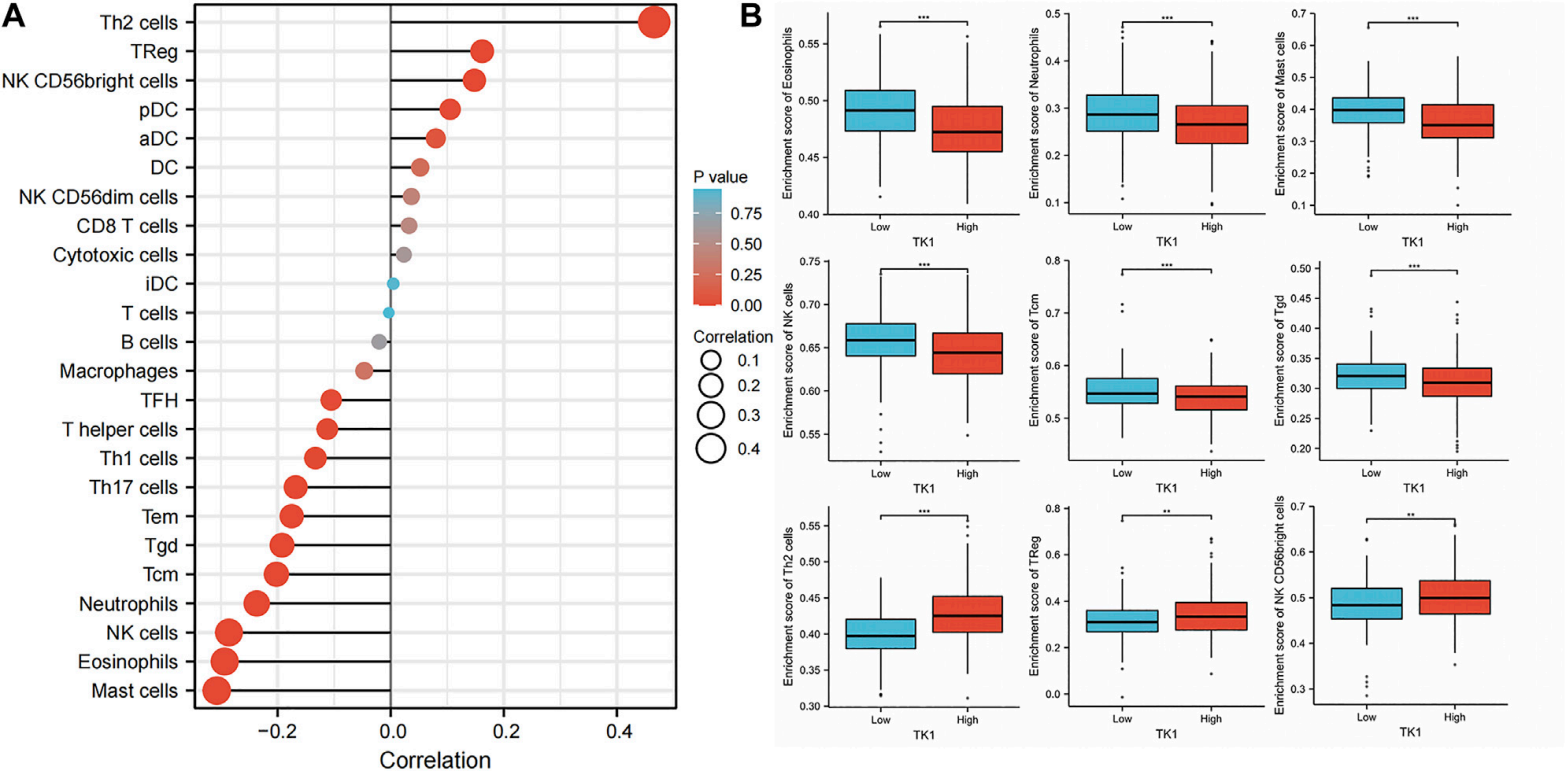
Effect of immune cell invasion on TK1 expression in individuals with PCa. **(A)** Relationship between the invasion contents of 24 immune cell types and the expression profile of TK1. **(B)** Comparison of the most associated levels of immune cell invasion in the high and low TK1 expression groups, including eosinophils, Tcm, neutrophils, mast cells, Tgd, NK cells, Th2 cells, TRed cells, and NK CD56bright cells. (*<0.05, **<0.01, ***<0.001).

The infiltration of 24 types of immune cells into PCa was first determined using the ssGSEA method, followed by a Spearman analysis to assess the relationship between TK1 with immune cell invasion (Figure 7A). Th2 cells (r = 0.466, *p* = 0.001), TReg cells (r = 0.162, *p* = 0.001), and NK CD56bright cells (r = 0.148, *p* = 0.05) were all positively linked to TK1 contents. Nonetheless, Mast cells (r = -0.307, *p* = 0.001), eosinophils (r = -0.294, *p* = 0.001), NK cells (r = -0.286, *p* = 0.001), Neutrophils (r = -0.237, *p* = 0.001), Tcm (r = -0.202, *p* = 0.001), Tgd (r = -0.192, *p* = 0.001) (Figure 7B). In addition, TK1 was remarkably linked to the gene signatures of monocytes, M1 macrophages, dendritic cells, Th1 cells, Th2 cells, and Tregs (Table 4).

**TABLE 4:** The correlations between TK1 and gene markers of immune cells in PCa and normal cells by TIMER.

Description	Gene markers	Nonecor	*p* Value	Puritycor	*p* Value
CD8+T cell	CD8A	-0.039	0.383	-0.010	0.832
	CD8B	-0.009	0.842	-0.011	0.830
T cell (general)	CD3D	0.062	0.165	0.088	0.074
	CD3E	0.030	0.510	0.052	0.285
	CD2	0.060	0.184	0.091	0.063
B cell	CD19	0.044	0.324	0.050	0.312
	CD79A	0.141	0.216	0.129	0.274
Monocyte	CD86	-0.001	0.997	-0.031	0.794
	CD115(CSF1R)	-0.206	*	-0.234	*
TAM	CCL2	-0.086	0.451	-0.076	0.520
	CD68	0.144	0.206	0.118	0.314
	IL10	0.037	0.748	0.040	0.733
M1 macrophage	INOS(NOS2)	0.083	0.469	0.070	0.551
	IRF5	-0.184	0.105	-0.213	*
	COX2(PTGS2)	0.215	*	0.231	*
M2 macrophage	CD163	-0.080	0.484	-0.081	0.491
	VSIG4	-0.058	0.613	-0.076	0.521
	MS4A4A	-0.099	0.384	-0.104	0.377
Neutrophils	CD66b (CEACAM8)	0.188	*	0.186	0.112
	CD11b (ITGAM)	-0.121	0.286	-0.155	0.187
	CCR7	-0.162	0.153	-0.172	0.142
NK	KIR2DL1	0.058	0.612	0.008	0.949
	KIR2DL3	-0.058	0.614	-0.039	0.739
	KIR2DL4	0.181	0.111	0.165	0.161
	KIR3DL1	-0.098	0.392	-0.086	0.465
	KIR3DL2	0.021	0.855	-0.020	0.864
	KIR3DL3	0.003	0.979	0.046	0.697
	KIR2DS4	-0.077	0.502	-0.077	0.516
Dendritic cell	HLA-DPB1	-0.149	0.189	-0.146	0.213
	HLA-DQB1	-0.102	0.371	-0.098	0.407
	HLA-DRA	-0.188	*	-0.186	*
	BDCA-1(CD1C)	-0.214	0.058	-0.234	0.045
	BDCA-4(NRP1)	0.271	**	0.297	*
	CD11c	0.090	0.428	0.026	0.823
Th1	T-bet (TBX21)	-0.146	0.198	-0.128	0.277
	STAT4	0.010	0.927	0.026	0.824
	STAT1	0.310	***	0.355	***
	IFN-γ(IFNG)	-0.010	0.928	0.045	0.706
	TNF-α(TNF)	0.074	0.518	0.083	0.483
Th2	GATA3	0.311	***	0.350	***
	STAT6	-0.262	**	-0.250	**
	IL13	0.219	**	0.223	**
Tfh	BCL6	0.108	0.343	0.102	0.388
Th17	STAT3	0.178	0.116	0.202	0.085
	IL17A	0.178	0.116	0.183	0.119
Treg	FOXP3	0.145	0.202	0.136	0.246
	CCR8	0.032	0.781	0.024	0.836
	STAT5B	-0.117	0.302	-0.105	0.374
	TGFβ(TGFB1)	0.254	**	0.260	**
T cell exhaustion	PD-1(PDCD1)	-0.012	0.914	0.000	0.999
	CTLA4	-0.082	0.472	-0.096	0.415
	LAG3	0.185	0.102	0.197	0.093
	TIM3(HAVCR2)	0.043	0.708	0.016	0.892
	GZMB	0.063	0.580	0.052	0.658

*<0.05, **<0.01, ***<0.001.

### Association of TK1 methylation with immune infiltrates in PCa

**FIGURE 8:**
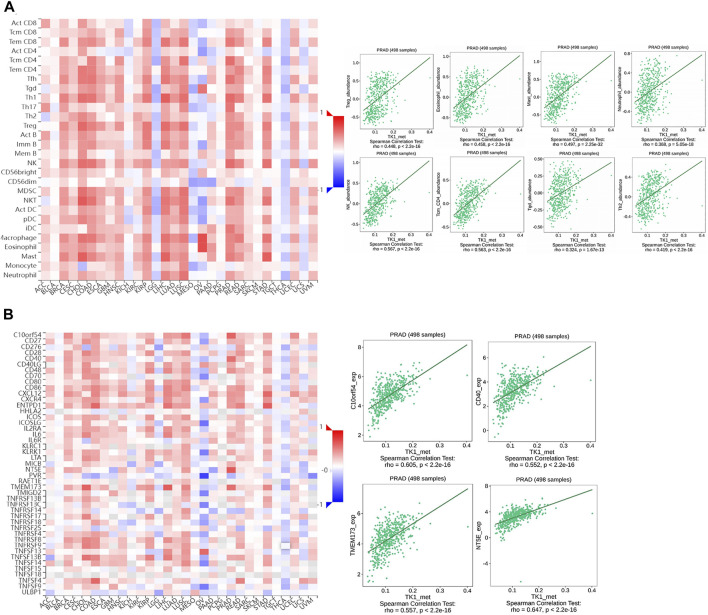
The relationship of the methylation status of TK1 with immune infiltration in PCa. **(A)** Relationship of the methylation status of TK1 with Th2 cells, Tregs, NK eosinophils, NK cells, neutrophils, Tcm, and Tgd in PCa available from the TISIDB data resource. **(B)** Relationship of the methylation status of TK1 with immunostimulator PCa available from the TISIDB data resource.

To assess the effect of TK1 methylation on PCa progression, we evaluated the relationship between TK1 methylation and immune cell invasion using the TISIDB platform. The results showed that TK1 methylation status correlated with Th2 cells (r = 0.418, *p* = 2.2e-16), TReg cells (r = 0.448, *p* = 2.2e-16), mast cells (r = 0.497, *p* = 2.25e-32), eosinophils (r = 0.458, *p* = 2.2e-16), NK cells (r = 0.567, *p* = 2.2e-16), Tcm cells (r = 0.563, *p* = 2.2e-16), and Tgd cells (r = 0.324, *p* = 1.67e-13) (Figure 8A). Similarly, the methylation status of TK1 was also linked to immunostimulants, such as C10orf54 (r = 0.605, *p* = 2.2e-16), CD40 (r = 0.552, *p* = 2.2e-16), TMEM173 (r = 0.557, *p* = 2.2e-16), and NT5E (r = 0.647, *p* = 2.2e-16) (Figure 7B).

### Drug responses related to TK1 expression in PCa

**FIGURE 9:**
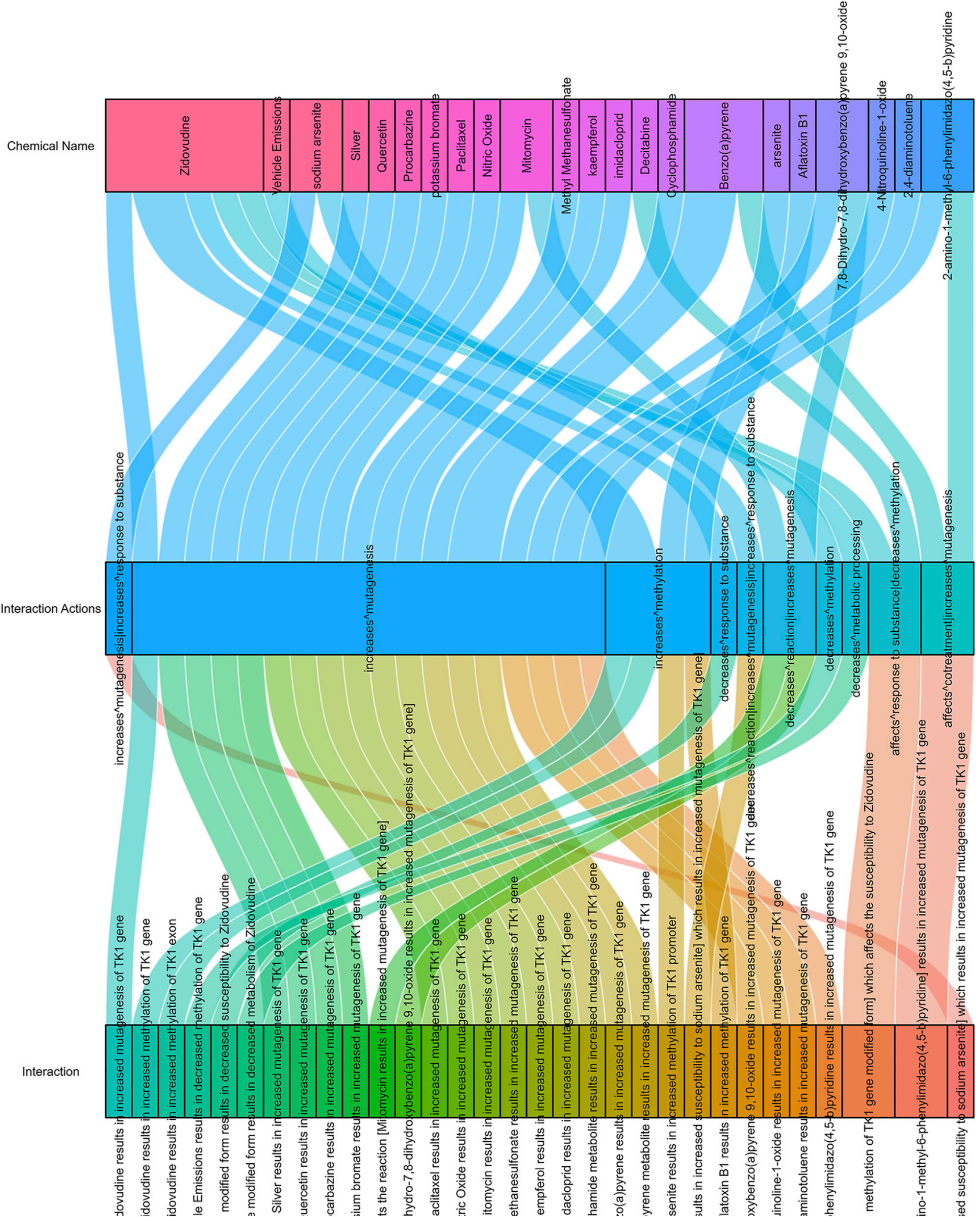
Drug response related to TK1 expression in PCa.

As shown in Figure 9, there are 38 drugs that are helpful in the treatment of prostate cancer with high TK1 expression (two drugs can affect TK1 cotreatment, 24 drugs can increase TK1’s mutagenesis, two drugs affect the response to the substance of TK1, three drugs decrease TK1 reaction, one drug increase the response to TK1, four drugs can increase TK1 methylation, and one drug decrease metabolic processing).

## Discussion

PCa is the most commen cancer threat in men, and biochemical failure in a short time after radical treatment is linked to a high risk of tumor progression (Hernandez et al., 2019). Thymidine kinase 1 (TK1) is a key enzyme involved in synthesis DNA precursors. It is upregulated in the S phase of the cell cycle, and its presence in cells is an indicator of active cell multiplication (Chen et al., 2010). TK1 levels are dramatically increased when DNA is damaged by radiation or chemotherapy drugs (Haveman et al., 2006; Chen et al., 2010). This may improve the restoration efficiency of thymidine released during DNA breakdown. TK1 is involved in DNA repair, and the impairment or low expression of TK1 during DNA restoration may lead to cell death (Chen et al., 2010). Collectively, these findings reveal that TK1 may be involved in the development of PCa in various ways and that repressing its functional site may be a novel approach for treating PCa.

Herein, the contents of TK1 in PCa and its relevance to the diagnosis, along with the prognosis of PCa, came to light. TK1 is dysregulated in several cancer types and plays a vital role in cancer onset and progression (Topolcan & Holubec, 2008). Based on TCGA data analysis, PCa shows remarkably high TK1 mRNA expression. Sun R. et al. Reported that TK1 expression and activity are affected by oxidative stress and nucleoside analog treatment (Sun et al., 2020). Researchers have also proven that the TK1 gene promoter’s abnormal CpG island methylation is involved in controlling TK1 expression in failing and non-failing human left ventricular myocardium (Koczor et al., 2013a).

Consequently, we then explored the promoter methylation levels of TK1 in PCa. We found that the DNA methylation level of TK1 in cancer tissues was markedly lower than that in non-tumorous tissues, which may suggests that low TK1 promoter methylation levels are due to the overexpression of TK1 in PCa. In addition, we found that TK1 expression was remarkably positively linked to the expression of the E2F gene family in PCa, with E2F1/2/3/5 being highly expressed in PCa. Several prior studies have shown that the transcription factor E2F1 can directly regulate TK1 and promote tumor proliferation (Zhu et al., 2018). We speculate that E2F2/3/5 have a similar effects.

Recently, it was shown that despite being a biomarker, TK1 also participates in cancer invasion and progression (Tilli et al., 2016; Alshabi et al., 2019; Song et al., 2019). TK1 has been previously documented to enhance cancer progression, and high TK1 levels are predictive of poor prognosis in individuals with cancer. To determine whether TK1 is an independent predictive factor for PCa, we studied the prognosis of patients with PCa with diverse TK1 levels. We found that high a TK1 content in PCa tissues was linked to poor prognosis in individuals with PCa. A multivariate Cox regression further supports that TK1 overexpression is a factor of poor prognosis in PCa patients, showing that TK1 is a prognostic biomarker for PCa. Genes in the same subpopulation tend to be coexpressed and synergistically coregulated.

To reveal the biological function of TK1, we performed co-expression and functional enrichment assessments. In addition, to investigate the function of TK1 in PCa, we performed a single-cell analysis. The results revealed that TK1 primarily participates in the cell cycle, DNA repair, and apoptosis. The dynamic crosstalk of tumor cells with the TME may trigger a chronic inflammatory environment that drives the onset and progression of cancer (Fridlender et al., 2009; Mehla & Singh, 2019).

To establish the role of TK1 in the TME, the relationship between TK1 and immune invasion in PCa was investigated. Our findings suggest that TK1 expression positively correlates with the immune invasion of immune cell populations consisting of Th2 cells, Treg cells, and NK CD56bright cells. However, TK1 expression was negatively linked to mast cells, eosinophils, NK cells, neutrophils, Tcm, and Tgd. The relationship between TK1 levels and genetic markers of immune cells further showed that TK1 interacts with Th2 and Treg cells. In the PCa microenvironment of PCa, high levels of Treg cells were associated with poor prognosis (Shen et al., 2021). These data suggest that TK1 may have a sophisticated modulatory role in immune-related processes.

The dysregulation of DNA methylation in the epigenome affects the immunogenicity of tumors and immune cells in the TME (Hogg et al., 2020). Our study found that the hypomethylation status of TK1 tended to be more common in high-grade and advanced PCa, which may illustrate that a change in TK1 methylation patterns inhibits PCa progression. The possible reason for this may be because the hypermethylation status of TK1 is detrimental to cell proliferation (Koczor et al., 2013b). To determine the mechanism by which TK1 methylation inhibits PCa progression, we explored the relationship between TK1 methylation and immune invasion. Our findings illustrated that TK1 methylation was positively linked to immune cells and immunostimulatory factors. The hypermethylation status of TK1 may contribute to the inhibition of tumor progression in PCa, which helps explain the hypomethylation status of TK1 in PCa. Methylation of TK1 may indicate cancer immune infiltration and response to immunotherapeutic agents in patients with PCa. It may be a potential predictor of PCa patients’ response to immunotherapeutic agents. The methylation level of TK1 was shown to act as a valid prognostic biomarker for PCa.

Based on clinical and pathophysiological data, TK1 is a promising therapeutic target for PCa. There are 38 drugs that are useful for treating PCa with high TK1 expression. Interestingly, we found that multiple drugs acted on TK1 methylation. Therefore, we hypothesize that the PCa development could be blocked by drugs that induce TK1 hypermethylation.

In summary, this study showed that TK1 is a prognostically relevant marker for PCa. TK1 expression and methylation status are linked to immune cell invasion and immune modulation. However, this study had certain limitations. First, gene expression analysis based on open source data resources may have limited accuracy. This possibility requires further investigation using *in vitro* and *in vivo* models to assess TK1 and the possible biological mechanisms of tumor immune crosstalk in PCa. Second, the impact of the E2F family targets on TK1 need to be further explored. Third, the TK1 gene methylation changes that occur during PCa development need to be further investigated. In summary, our study highlights a novel immunomodulatory function of TK1 hypermethylation in PCa.

## Data Availability

The original contributions presented in the study are included in the article/supplementary material, further inquiries can be directed to the corresponding author.
